# Climate influences on female survival in a declining population of southern elephant seals (*Mirounga leonina*)

**DOI:** 10.1002/ece3.7919

**Published:** 2021-07-27

**Authors:** Sophia Volzke, Clive R. McMahon, Mark A. Hindell, Harry R. Burton, Simon J. Wotherspoon

**Affiliations:** ^1^ Institute for Marine & Antarctic Studies University of Tasmania Hobart Tas. Australia; ^2^ IMOS Animal Tagging Sydney Institute of Marine Science Mosman NSW Australia; ^3^ Antarctic Climate and Ecosystems Cooperative Research Centre University of Tasmania Hobart Tas. Australia; ^4^ Australian Antarctic Division Department of Agriculture, Water and the Environment Kingston Tas. Australia

**Keywords:** capture‐mark‐recapture studies, demographics, ecological modeling, El Niño Southern Oscillation, Hidden Markov Model, population dynamics, vital rates

## Abstract

The Southern Ocean has been disproportionately affected by climate change and is therefore an ideal place to study the influence of changing environmental conditions on ecosystems. Changes in the demography of predator populations are indicators of broader shifts in food web structure, but long‐term data are required to study these effects. Southern elephant seals (*Mirounga leonina*) from Macquarie Island have consistently decreased in population size while all other major populations across the Southern Ocean have recently stabilized or are increasing. Two long‐term mark‐recapture studies (1956–1967 and 1993–2009) have monitored this population, which provides an opportunity to investigate demographic performance over a range of climatic conditions. Using a 9‐state matrix population model, we estimated climate influences on female survival by incorporating two major climatic indices into our model: The Southern Annular Mode (SAM) and the Southern Oscillation Index (SOI). Our best model included a 1 year lagged effect of SAM and an unlagged SOI as covariates. A positive relationship with SAM1 (lagged) related the previous year's SAM with juvenile survival, potentially due to changes in local prey availability surrounding Macquarie Island. The unlagged SOI had a negative effect on both juvenile and adult seals, indicating that sea ice dynamics and access to foraging grounds on the East Antarctic continental shelf could explain the different contributions of ENSO events on the survival of females in this population.

## Graphical abstract

This research investigated the influence of large‐scale climate modes (SAM and SOI) on female southern elephant seal survival (φ) by creating demographic models from historic capture‐mark‐recapture data. Map of the Southern Ocean from Tasmania (top) to East Antarctica and the Ross Sea (bottom) with Macquarie Island location marked by a small circle. Surrounding Antarctica, the Polar Front (blue line), 60th parallel south, (gray circle) and winter sea ice extent (white) are overlaid. Top right: Vegetation map of Macquarie Island (adapted from the Australian Antarctic Data Centre map no. 13106) and stylized illustration of a female southern elephant seal, *Mirounga leonina* (artwork by: Sophia Volzke).

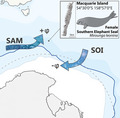

## 1 INTRODUCTION

Global climate change is affecting many plant and animal populations and is altering ecosystem structure and function. Climate influences many aspects of natural systems including animal distributions, habitat conditions, and food web structure (Hughes, 2000). Therefore, ecosystem responses to climate change are diverse and often indirect and difficult to observe (Weimerskirch et al., 2003). Quantifying how climate change affects animal populations requires information linking external environmental conditions with vital rates, particularly survival, and fecundity.

The Southern Ocean has been disproportionally affected by climate change (Macdonald & Wunsch, 1996) making it an ideal system for quantifying the links between climate and animal vital rates (Trathan et al., 2007). Southern Ocean food webs are relatively simple (Ainley & DeMaster, 1990), and consequently, the transition functions from primary producers to top predators can be captured realistically by ecological models (New et al., 2014). The species that inhabit the high latitudes (beyond 60°S) have evolved specialized traits to cope with the unique habitat conditions in cold but stable water temperatures (Rogers, 2008). However, these species are increasingly subjected to a rapidly changing environment (Constable et al., 2014; Hughes, 2000; Rogers et al., 2020). Understanding how these changes affect species and the wider ecosystem is challenging given the elusive nature of marine animals and general limitations of monitoring processes underwater (Hazen et al., 2019). Long‐term datasets are essential to detecting climate‐induced changes in habitat conditions over time, including atmospheric warming, sea‐level rise, and other environmental signals (Barbraud & Weimerskirch, 2001; Smith et al., 1999).

Long‐term census data exist for several predator species in the Southern Ocean (Hindell et al., 2003; Weimerskirch et al., 2003). Changes in predator abundance can indicate flow‐on effects on the local food web structure, which can act as a warning signal for broader shifts in ecosystem composition (Horn & Whitcombe, 2015). To quantify which traits affect population growth rates, ecologists must consider the interactions between individual processes (*e.g*., survival, fecundity, and recruitment) and external influences (*e.g*., habitat conditions, climate, and species interactions). Long‐lived marine predators are frequently the subject of demographic studies because larger species are relatively easy to capture and many return to breeding grounds seasonally in large numbers where monitoring projects can be established (Hazen et al., 2019). In the Southern Ocean, the demography of penguins, seals, and seabirds has been used to investigate the influence of climate change on ecosystems (Barbraud & Weimerskirch, 2001; Constable et al., 2014; Hughes, 2000; Weimerskirch et al., 2003). However, predators and their prey species can also adapt, to some extent, to changing habitat conditions (Guinet et al., 2014). Additionally, broad‐scale regime shifts must be distinguished from localized effects that may only be relevant in specific habitats (Weimerskirch et al., 2003). Due to many of these complex interactive components, population trajectories of Southern Ocean species under changing climatic conditions remain poorly described and understood (Constable et al., 2014).

Southern elephant seals (*Mirounga leonina*) are large, highly polygynous capital breeding mammals (Le Boeuf & Laws, 1994). Their circumpolar distribution reflects climate signals from across vast areas of the ocean for which long‐term vital rates observations are available (Desprez et al., 2018; Hindell et al., 2016; McMahon & Burton, 2005; Oosthuizen et al., 2019). Two population monitoring projects from Macquarie Island have provided extensive capture–mark–recapture histories for this species (Carrick & Ingham, 1962; Hindell, 1991; McMahon et al., 2000). This includes monitoring programs dating back to the mid‐20th century when many Southern Ocean mammal species were recovering from commercial harvesting (Carrick et al., 1962; Hindell & Burton, 1988b; Tønnessen & Johnsen, 1982). The Macquarie Island population of southern elephant seals is the only breeding population in the Pacific Ocean section of the Southern Ocean and has been continuously declining since the 1960s (van den Hoff et al., 2014). The other three genetically distinct subpopulations of the species (Corrigan et al., 2016) have since stabilized or are even increasing (Hindell et al., 2017b). Interspecific competition for food resources and the influence of climate change on the foraging habitat are the most likely causes of the ongoing decline at Macquarie Island (Hindell et al., 2017b; McMahon et al., 2005). However, elucidating the underlying mechanisms has so far been limited to describing correlative linear relationships between environmental change and elephant seal demography (*e.g*., van den Hoff et al., 2014; McMahon et al., 2017). The proximate demographic processes are still unclear.

Statistical approaches to analyze capture–mark–recapture data have developed rapidly, aided by technological advances and computing capacity (Gelfand et al., 2019). Matrix Population Models (MPMs) can be fitted to capture–mark–recapture data by incorporating the life‐history stages of a species with age or event‐specific states (Caswell, 2001). These models use Markov Chain Monte Carlo algorithms or Hidden Markov Models, enabling the analysis of incomplete capture histories to estimate complex survival probabilities, which may vary with individual characteristics (age, sex) and biotic (habitat, competition) or abiotic (environmental) factors (Zucchini et al., 2016). Previous research has optimized the model structure for elephant seal MPMs by incorporating relevant life‐history stages and the breeding ecology for this species (Desprez et al., 2013, 2014, 2018). However, the influence of external environmental covariates on elephant seal demography has not yet been investigated with these models.

This work quantified the influence of climate on southern elephant seal demography by developing matrix population models which incorporate two important climatic drivers of the Southern Ocean: The Southern Annular Mode (SAM) and Southern Oscillation Index (SOI). This expands on previous research into the ongoing population decline at Macquarie Island, giving novel insights into the contribution of each climatic variable on the survival of female southern elephant seals over time. Previous research considered individual climatic influences on adult female seals only (van den Hoff et al., 2014; McMahon et al., 2017), while most investigations into the population decline at Macquarie Island focused on juvenile survival and recruitment (Desprez et al., 2014; McMahon et al., 2000, 2015). Incorporating the life history of the species into a state‐based matrix population model enables the complex analysis of different life stages in one model (including juvenile, subadult, and adults). We expect climatic influences to have different effects on different age classes because juveniles and adults feed in different locations, which respond differently to climatic indices (Field et al., 2005; Lovenduski & Gruber, 2005).

## 2 METHODS

### 2.1 Data collection

Macquarie Island (54°30′ S, 158°57′ E) lies 1,500 km southeast of Tasmania and 1,300 km to the north of the Antarctic continent. The island is the only breeding location for southern elephant seals within the Pacific sector of the Southern Ocean (McMahon et al., 2005) and is a closed breeding population (Corrigan et al., 2016; Slade et al., 1998). There were 49 annual censuses of the breeding population from 1949–2015 to monitor the population trends on Macquarie Island (Hindell et al., 2017a). Over two periods (1956–1967 and 1993–1999), a subset of pups born on the isthmus was marked permanently to provide longitudinal individual demographic data (Carrick et al., 1962; Carrick & Ingham, 1962; Hindell, 1991; McMahon et al., 1999).

Elephant seal pups are considered to wean when they leave the breeding harems (McMahon et al., 2000). Between 1951–1965 (Hindell, 1991) and 1993–1999 (McMahon & Burton, 2005), weaners were captured and marked permanently by hot iron branding. This method provides a lifetime individual mark (Hindell & Little, 1988), that does not affect survival (McMahon et al., 2006). The relevant permits were obtained from the Australian Antarctic Animal Ethics Committee and the Tasmanian Parks and Wildlife Service. Marked seals were then followed through time by conducting daily resights of the island isthmus and opportunistic monthly searches of the entire island beaches from 1993–2001 (McMahon & Burton, 2005). For the earlier dataset, search efforts varied between organized searches of the entire island (1952 and 1959) and opportunistic sightings recorded anytime between 1952 and 1979 (Hindell, 1991).

### 2.2 Data handling

The demographic data were collected during two study periods with capture‐mark‐recapture efforts: the 1960s (1957–1979) and 1990s (1993–2003). Outside of those, a small number of ad‐hoc sightings of marked seals from the 1980s and early 2000s were also included. Capture–mark–recapture analysis requires a master sex recorded with each unique seal identifier to distinguish male from female individuals. Weaned seals were identified as male or female during their first capture upon branding. Each assigned master sex was cross‐checked with re‐sightings of the same individuals in later life stages if available. The extreme sexual dimorphism of the species makes it easy to distinguish adult bulls from breeding females (Le Boeuf & Laws, 1994). A few individuals with inconsistent age classes were removed, as well as any seals first tagged as adults, as these do not fit within a model based on developmental life histories. The resulting number of individuals used in this dataset was 9,690 female seals (Appendix S1A). We defined the elephant seal annual cycle as beginning in September (the start of the breeding season) and ending in August the following year. The bulk of the resight effort occurred during the breeding season, when the animals are hauled up on land in large numbers (Hindell & Burton, 1988a). Any adult females resighted during September and October were assumed to be breeding (Desprez et al., 2018).

### 2.3 Demographic modeling

Female southern elephant seal survival was modeled using Matrix Population Models, which incorporated developmental life‐history stages (Caswell, 2001). This was done using a Hidden Markov Model (HMM), which enabled the incorporation of incomplete capture histories due to missed observations in the data (Pradel, 2005). Previous research on this population found that survival probability estimates are greatly improved by including a first‐time breeder state and accounting for nonbreeding adults that are seen outside of the breeding season (Desprez et al., 2014, 2018). This forms a baseline for our model state structure.

The developmental stages of female southern elephant seals were juveniles (*J*), first‐time breeders (*FB*), breeding adults (*B*), and dead (*D*) (Figure 1.1). Once marked, all newly weaned seals were automatically assigned to the juvenile state. The juvenile state was divided into 6 substates *J0, J1, J2, J3, J4, J5*. This allowed the incorporation of different maturing probabilities (*M_x_*) according to the age of the individual. The transition matrix included all states and juvenile substates (Figure 1.2). Female elephant seals mature between the ages of 3–7 years (Desprez et al., 2014), which was defined as the first reproductive attempt of the individual, regardless of the success. Maturing seals were allocated to the first‐time breeder state (*FB*), from which they automatically matured to become breeding adults (*B*) the following year. Including the FB state significantly improved survival estimates for adult female seals (Desprez et al., 2014). In the emission matrix (*i.e*., the recapture probability matrix) (Figure 1.3), adult female elephant seals resighted during the breeding season (September to October) (seen breeding *d_B,t_*) were distinguished from adults observed any other time of the year (seen other *d_N,t_*). Incorporating this additional emission category captured the possibility of adult female elephant seals skipping a breeding event (Desprez et al., 2018). This is relevant because these “nonbreeders” were resighted and recognized to be alive, which improved survival estimates, but they did not contribute to breeding and recruitment for that season. All emission (detection) probabilities were modeled as time‐dependent.

**FIGURE 1:**
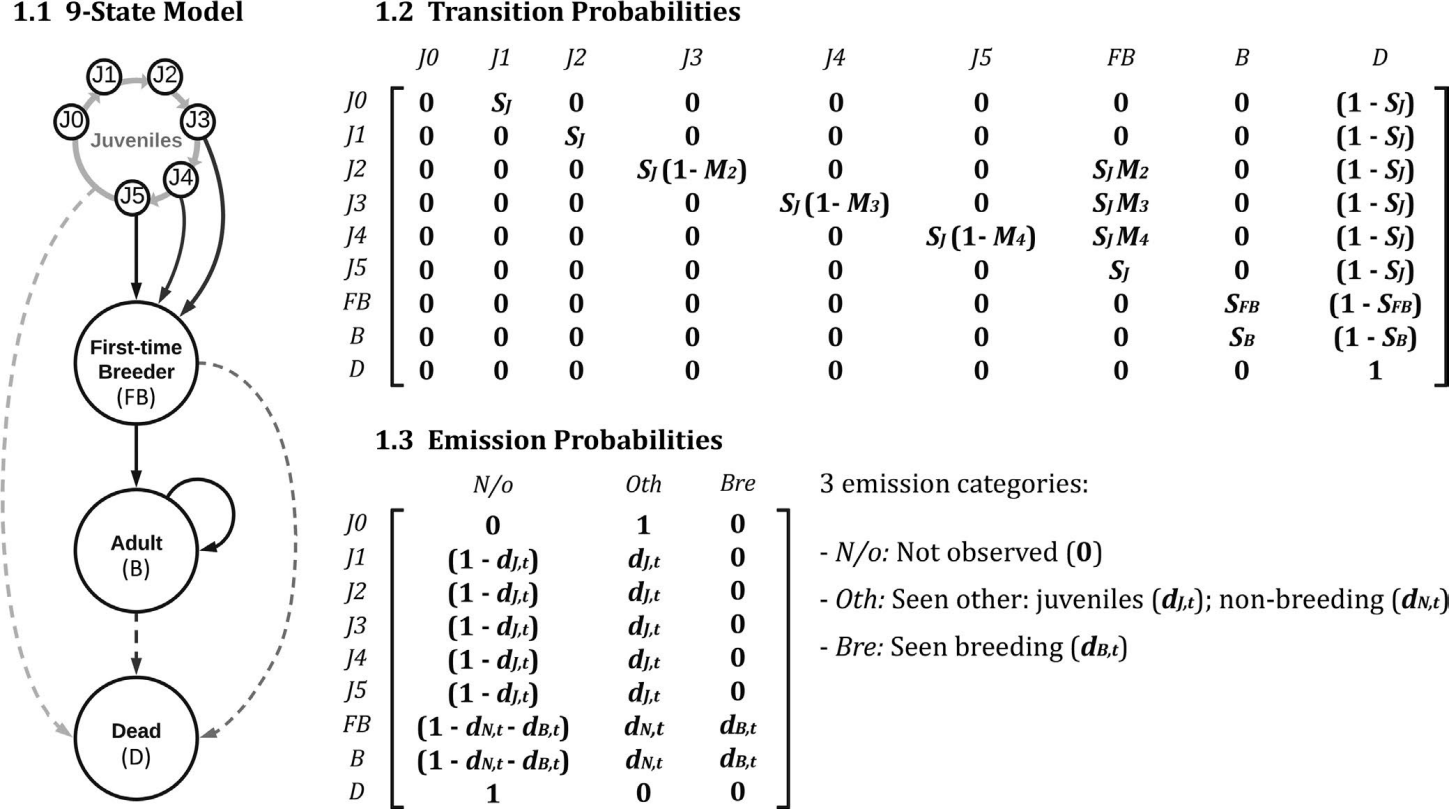
The 9‐State Matrix Population Model used to analyze the survival of southern elephant seals from Macquarie Island. 1.1. Schematic representation of the model. Circles represent states, and lines illustrate transition probabilities as marked. A looping arrow on the breeding adult state means seals can be allocated to this state for more than one resight event. 1.2. Transition Matrix of probabilities. Each state (*J0‐J5, FB, B, D*) corresponds to one row and column in the matrix. Moving from one state to the next is represented by transition probabilities, which are denoted as *S_x_* (survival) *M_x_* (maturing). 1.3. The Emission Matrix includes probabilities of observing an individual (*d_x_*) in each state, which is modeled as time‐dependent (*t*). Rows are labeled in order of developmental stages and columns represent three emission categories, as listed to the right of the matrix

### 2.4 Climate covariates

Two climate indices were incorporated into the models as covariates. These were derived from atmospheric pressure measurements representing the two major climate indices of the Southern Ocean climate system (Rogers et al., 2020; Simpkins et al., 2012): The Southern Annular Mode (SAM) (Karoly, 1990) and Southern Oscillation Index (SOI) (Karoly, 1989). Both were averaged into annual means from monthly data, which is calculated from October to September each year to correspond with the elephant seal life cycle. The SAM influences wind patterns in the Southern Ocean (Hall & Visbeck, 2002) and is calculated from zonal pressure differences between the latitudes of 40°S and 65°S (Marshall & National Center for Atmospheric Research Staff, 2018). SAM records are available online from 1957 at Marshall and National Center for Atmospheric Research Staff (2018). The SOI describes anomalies associated with the ENSO cycle (El Niño Southern Oscillation) in the tropics (McPhaden et al., 2006). Various forms of this index exist; we used standardized sea‐level pressure data (NOAA Climate Prediction Center, 2015), which is available from 1951. Both covariates were checked for intercorrelation with Pearson's correlation coefficient (Appendix S1B). Previous research has highlighted the importance of a temporal lag when analyzing environmental covariates (van den Hoff et al., 2014). Depending on the demographic parameter of interest, lagged models should allow an appropriate amount of time for the external influence to be detectable in population dynamics (Weimerskirch et al., 2003). For example, van den Hoff et al. (2014) concluded that recruitment to the breeding colony is best modeled with a lag of 3 years, which is the youngest age at which elephant seal pups can reach maturity (McMahon et al., 2003). Our research question concentrates on survival, which arguably would only take up to 1 year to take hold in the population if, for example, food availability in the preceding year had biological consequences. Thus, models were created with both SAM and SOI and with a lag of 1 year, where SAM1 and SOI1 correspond to the SAM and SOI conditions of the previous year. Nine models with different combinations of lagged and unlagged SAM and SOI were compared. Additionally, we also tested one plain model without covariates (Table 1). Although resight data and SAM values exist from 1957 onwards, the first year of sightings was excluded so that both lagged and unlagged covariate models could be compared by Akaike information criterion (AIC) for model selection (Burnham & Anderson, 2004).

**TABLE 1:** Model selection results comparing various combinations of environmental covariates to explain residual variability in the survival of southern elephant seals (*Mirounga leonina*) at Macquarie Island

Model	SAM	SOI	SAM1	SOI1	Log likelihood	# Var	AIC	Δ AIC
	(No Lag)		(1 Year Lag)					
SPLINE w SAM1 & SOI		+	+		−35039.10	9	70,096.20	0.00
SPLINE w SAM & SOI	+	+			−35041.18	9	70,100.36	4.16
SPLINE w SAM1 & SOI1			+	+	−35042.75	9	70,103.50	7.30
SPLINE w SAM & SOI1	+			+	−35043.35	9	70,104.70	8.50
SPLINE w SAM & SOI + SAM1 & SOI1	+	+	+	+	−35037.85	15	70,105.70	9.50
SPLINE w SOI only		+			−35046.84	6	70,105.68	9.48
SPLINE w SAM only	+				−35047.53	6	70,107.06	10.86
SPLINE w SOI1 only				+	−35050.36	6	70,112.72	16.52
Plain SPLINE					−35053.14	3	70,112.28	16.08
SPLINE w SAM1			+		−35061.36	6	70,134.72	38.52

Note

Mark and recapture data were fitted to a 9‐state spline matrix population model representing developmental life‐history stages of southern elephant seals. Environmental covariates were incorporated directly into the model, which include the seasonal averages for the Southern Annular Mode (SAM) (Marshall & National Center for Atmospheric Research Staff 2018) and Southern Oscillation Index (SOI) (NOAA Climate Prediction Center, 2015) from 1957 onwards. SAM1 and SOI1 represent the same data fit into the model with a lag of 1 year in relevance to the recorded resights. Models were ranked by Akaike information criterion (AIC) to determine the best fit.

### 2.5 Analysis

All analyses were performed in R 4.0.2 (R Core Team, 2020). The model was fitted by the Expectation–Maximization (EM) algorithm (Dempster et al., 1977), and standard errors and confidence intervals for the model parameters were estimated by parametric bootstrap (Efron, 1994). The log odds of survival of juveniles, first‐time breeders, and adults were modeled as linear combinations of the lagged and unlagged climate covariates, and a B‐spline matrix with equally spread knots (*df* = 15) was added to represent background survival varying slowly over time.

To fit the model by the EM algorithm, some reasonable initial parameter estimates were entered based on informed guesses. Scott's algorithm (Scott, 2002) then determined the expected number of individuals in each state for each year (weights). Emission probabilities for each year were estimated from the observed and expected states by maximum likelihood, and transition probabilities are estimated by fitting Binomial generalized linear models to the expected number of state transitions. Given these updated estimates of the model parameters, the expected number of individuals in each state by year was again determined by Scott's algorithm, and the entire process was iterated until convergence was achieved. Although the EM algorithm produced maximum likelihood estimates of the model parameters, as the models were fitted to the expected numbers in each state by year, the variability of the parameter estimates was likely underestimated. To derive reliable estimates of variability, standard errors and confidence intervals for the model parameters were estimated by parametric bootstrap. One hundred replicate data sets were simulated from the original fitted model, and standard errors and confidence intervals for the model parameters were based on the observed variability in the replicate fits. The bootstrapped simulated likelihoods were entirely consistent with observed likelihoods, thereby excluding the potential for overdispersion in the data (Appendix S1C). Model selection was performed only on the different combinations of the climate covariates. Models were ranked by Akaike information criterion (AIC) (Akaike, 1974) (Table 1).

## 3 RESULTS

The best model by AIC (Table 1) included SAM1 (SAM with a lag of 1 year) and SOI (no lag) as covariates.

### 3.1 Survival

**FIGURE 2:**
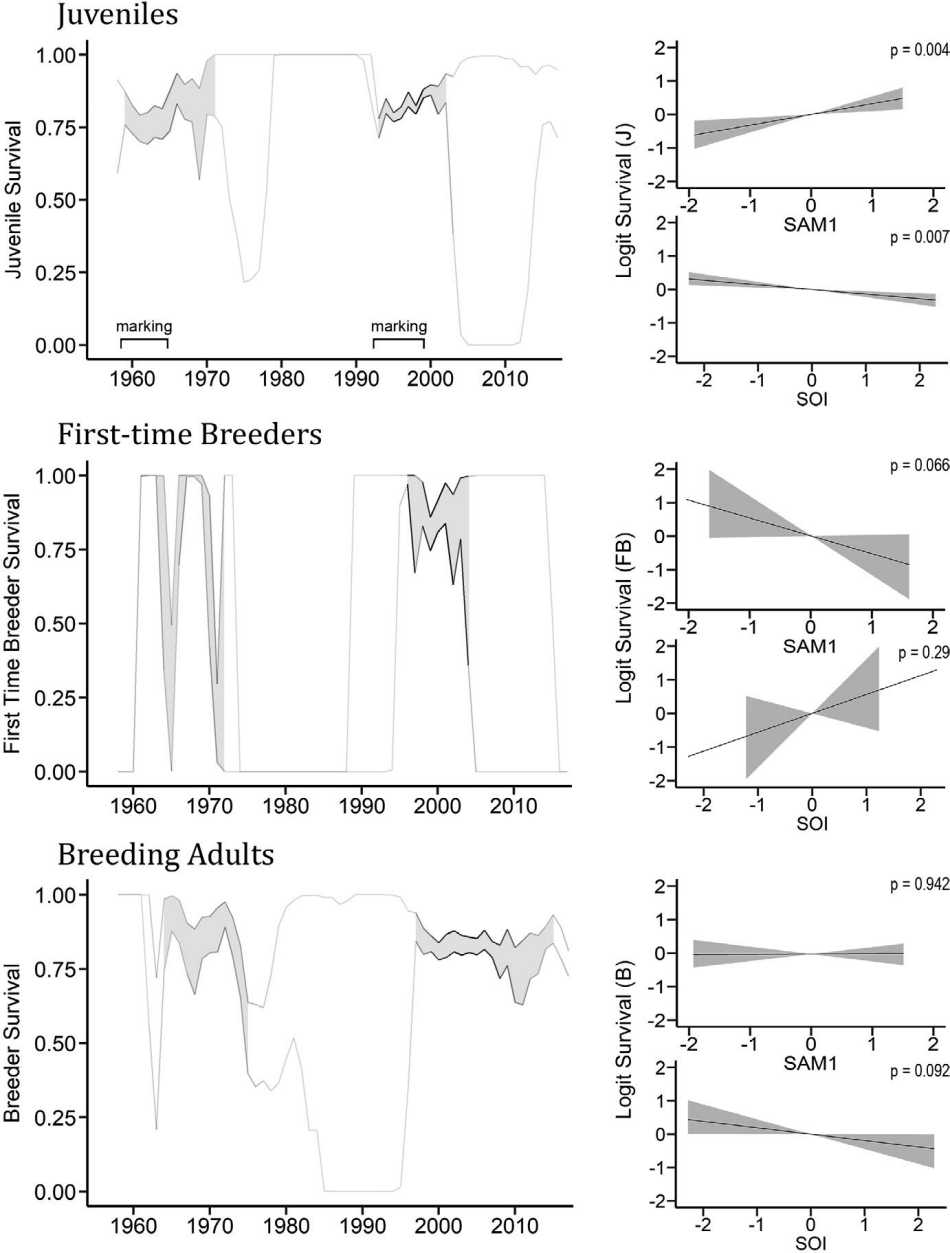
The survival of southern elephant seals at Macquarie Island from 1958 to 2015. Output from a 9‐state matrix population model, including two environmental covariates. Individual survival modeled over time (left) for juveniles (*J*), first‐time breeders (*FB*), and breeding adult (*B*) seals. Gradient outline from light gray to black indicates the estimated number of individuals in each state by year (weights). Shaded plots represent bootstrap quantiles (95%) with sufficient data present overall. The influence of environmental covariates on logit survival (right) is given for each group, including 95% confidence intervals in gray. SAM1 and SOI represent the annual averages (October ‐ September) for the Southern Annular Mode and Southern Oscillation Index respectively. The SAM is fitted in the model with a lag of 1 year

Survival probabilities for juvenile female southern elephant seals were similar between the two periods (average of 78% in the 1960s and 80% during the 1990s) (Figure 2). Juvenile survival had higher uncertainty around the 1970 breeding season. Figure 2 shows that the average survival is probably not lower; it is only the lower confidence band that is lower, possibly because of data limitations. Emission (detection) probabilities revealed a greater probability for detection of juveniles in the 1990s (around 60%) versus early 1960s (25%) (Appendix S2). Similarly, the probability for detecting breeding adults increased from an average 15% in the 1960s to 25% in the 1990s (see Appendix S2 for full emission and maturation probability results). The variability of the survival estimates was greater in the 1960s than the 1990s (Figure 2 Juveniles). Wider 95% confidence intervals (greater distance between quantiles) are seen overall for the 1960s and lighter shades in the curve outline represent a lower number of individuals in each state by year (weights) for this time period. First‐time breeder survival probabilities were less consistent (Figure 2 first‐time breeders). In the 1960s, *FB* survival probabilities were poorly estimated and jump erratically between 0 and 1, while in the 1990s estimates seem more stable in comparison but remain highly variable (between 0.5 and 1) (Figure 2). Breeding adult survival probability averaged 78% in the 1960s and 80% in the 1990s. As with the juveniles, the survival estimates for breeding adults were more variable in the earlier study period with less data underlining the curve outline of Figure 2. Model estimates were most precise between 2000–2010, when pups marked during the 1990s would have matured and been consistently resighted.

### 3.2 Climate influence on female survival

Annual averages of SAM and SOI were not correlated (Pearson *R* = −0.004, *p* = .97), and both terms were included in the models (Appendix S1B). The estimated effect of SAM1 and SOI on logit survival for each age class is shown in Figure 2. Both covariates contributed to juvenile survival. SAM1 showed a positive relationship with logit survival of juvenile seals (*β* = 0.32 [0.10; 0.54], *p* = .004), while SOI had a smaller negative effect (*β* = −0.14 [−0.23; −0.06], *p* = .007). First‐time breeders show no conclusive evidence for an effect of SOI on logit survival (*β* = 0.56 [−0.43; 1.61], *p* = .288). But SAM1 returned weak evidence for a negative relationship (*β* = −0.76 [−1.26; 0.03], *p* = .066). For adult seals, there was no evidence for a relationship between SAM1 and survival (*β* = 0.01 [−0.22; 0.21], *p* = .942). For SOI on the other hand, there was weak evidence for a negative effect on the survival of breeding adults. This relationship is of similar magnitude as the SOI’s effect on juvenile survival, but with greater uncertainty in estimates (*β* = −0.19 [−0.45; 0.00], *p* = .092).

**FIGURE 3:**
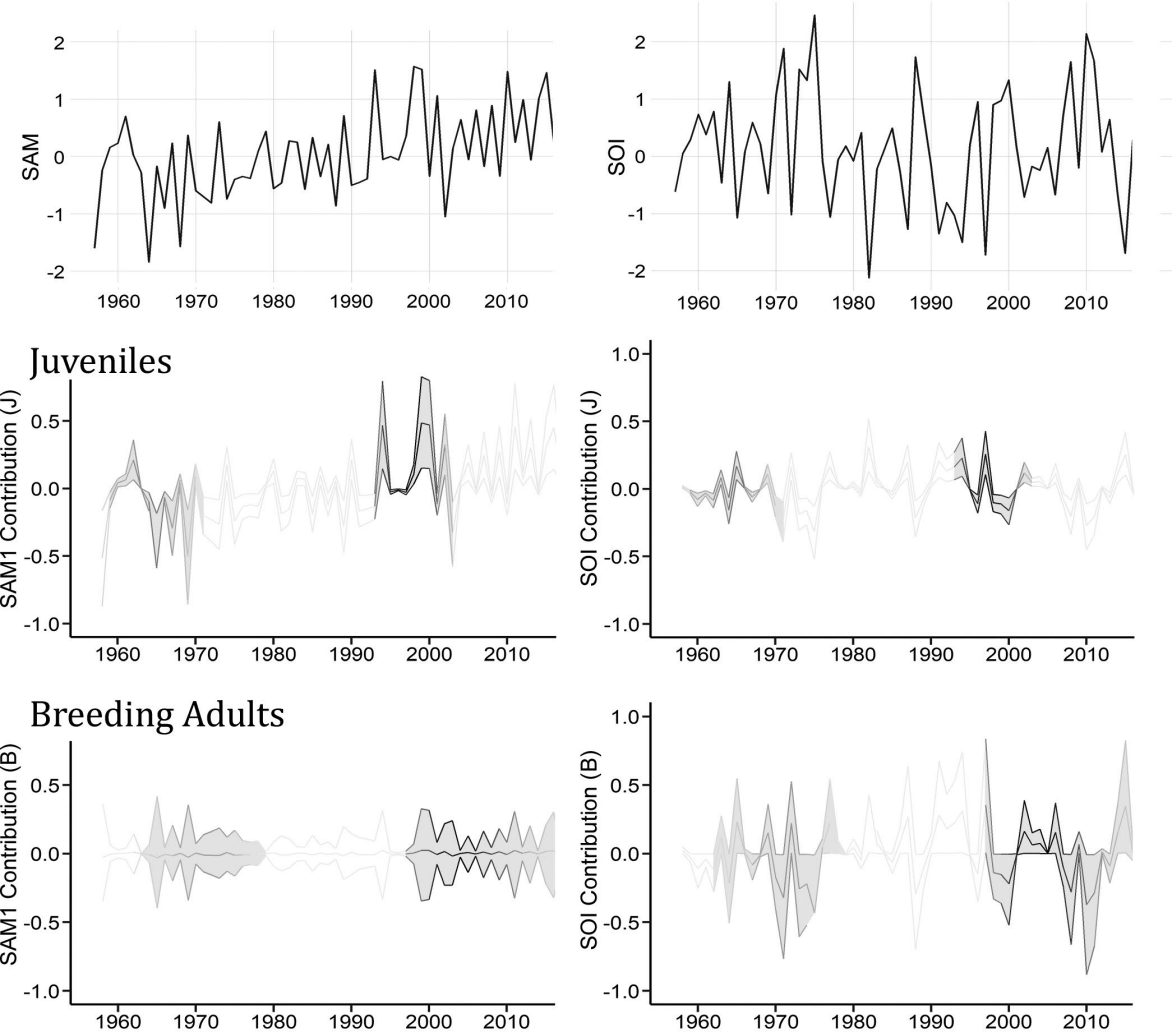
The influence of two environmental covariates on the logit survival of southern elephant seals over time. Annual averages (October–September) of SAM and SOI (top) are compared to the contribution of SAM1 and SOI on the survival of female southern elephant seals (*Mirounga leonina*) from 1960 to 2010 (middle and bottom). SAM1 is the Southern Annular Mode fitted to the data with a 1 ‐year lag in respect to resights. SOI represents the Southern Oscillation Index with no lag. Seal age classes (juveniles and breeding adults) are extracted from a 9‐state Matrix Population Model. Covariate influence is estimated by a Binomial GLM, calculated from 100 bootstrap iterations and plotted as 95% confidence intervals. Shaded plots highlight years with sufficient data present, as indicated by darker gradient outline for the estimated number of individuals in each state by year (weights)

Figure 3 illustrates the contribution of each covariate to logit survival over time for the three age classes. SAM has been slowly increasing over time, and correspondingly, the contribution of SAM1 in the 1960s was negative and positive in the 1990s. Specifically, the contribution of SAM1 to juvenile survival was up to 0.5 times greater in the 1990s. For breeding adults, the results were unclear with no detectable effect of SAM1 on survival (Figure 3: bottom left). In Figures 2 and 3, lighter shades in the graph outline in the 1960s indicate these estimates were based on a smaller number of individual seals. However, variance estimates (distance between quantiles) were largely consistent across both time periods of Figure 3.

The influence of SOI on logit survival was negative for juvenile female elephant seals. The tight confidence intervals around the SOI contribution (*J*) illustrate the robustness of this relationship (Figure 3); however, the magnitude of this effect is smaller relative to that observed for SAM1. There were no detectable differences between the 1960s and 1990s. La Niña years (positive SOI) result in a negative contribution to logit survival while El Niño events (negative SOI) result in a positive contribution. This negative relationship was more pronounced for breeding adults (Figure 3: bottom right); this corresponds to the SOI decreasing the odds of survival for breeding adult seals by as much as 0.75 times in 2010. It appears that the negative spikes in SOI contribution (*B*) were more extreme with a greater magnitude in the graph than the periods of positive contribution to logit survival of breeding adults.

## 4 DISCUSSION

We have demonstrated how climatic conditions influence the demography of a Southern Ocean predator. Our 9‐state matrix population model revealed how the survival of juvenile and adult female southern elephant seals at Macquarie Island is affected by two major climate indices: the Southern Annular Mode (SAM), which indicates the strength and position of strong westerly winds surrounding Antarctica (Hall & Visbeck, 2002; Karoly, 1990), and the Southern Oscillation Index (SOI), which measures pressure differences associated with El Niño and La Niña events in the Pacific Ocean (Karoly, 1989; McPhaden et al., 2006). Complex interactions with these two climatic drivers occur because SAM and SOI reflect changes in underwater current systems (Sallée et al., 2008), the location, and density of fronts (Sokolov & Rintoul, 2009), sea surface temperatures (Sen Gupta & England, 2007), mixed layer depths (Sen Gupta & England, 2006), and sea ice dynamics (Simpkins et al., 2012) in the Southern Ocean, all of which can influence biological elements of the ecosystem. We illustrated how climate can influence the demography of a large capital breeding animal population. Previous research concentrated on climatic influences on adult female elephant seals (van den Hoff et al., 2014; McMahon et al., 2017), while the analysis of the population decline at Macquarie Island focused on juvenile survival and recruitment (Desprez et al., 2014; McMahon et al., 2000, 2015). We present an important extension to this research by integrating the two approaches and examining climate effects on all age classes in one comprehensive demographic analysis.

A positive annual SAM index was associated with increased odds of survival for female juvenile elephant seals the following year by 0.3 times. Juveniles are defined as animals yet to breed and include seals making their first trip to sea. Conversely, a negative SAM index was associated with reduced juvenile survival the following year. However, breeding adults had inconclusive results for SAM1. The SOI was negatively related to the survival of both juveniles and breeding adults. A low (<1) SOI indicates El Niño years (McPhaden et al., 2006) was associated with an increased probability of survival of 0.15 times for juvenile and adult female elephant seals. In line with previous research (de Little et al., 2007; McMahon & Burton, 2005), La Niña years, indicated by high (>1) SOI values, reduced juvenile and adult female survival estimates. First‐time breeding seals were negatively related to SAM1, but this observation needs to be interpreted cautiously given the considerable uncertainty around this estimate. Parameters for the *FB* state are poorly estimated as they are derived from a single year in the seal's life history, while other states (juveniles and breeding adults) include data from multiple years. This lack of observational data for first‐time breeders, especially for the 1960s when the resight effort was lower, further highlights that information for this important life‐history state needs to be interpreted with some caution. Nonetheless, including this state in the model improved survival estimates for breeding adults. Young maturing elephant seals are not accustomed to the additional costs of reproduction and are also generally smaller with fewer fat reserves than experienced breeders (Desprez et al., 2014; Proaktor et al., 2007). The first reproductive event imposes a higher relative cost on survival for first‐time breeders, regardless of the age at which they mature (Desprez et al., 2014). Not including this as a separate state results in lower average survival estimates for adult seals, therefore, we like others (Desprez et al., 2014) separated first‐time breeders from experienced adults in the analyses.

As a lagged effect, SAM1 indicates that the biological conditions due to SAM take time to propagate up the food chain before influencing mesopelagic prey and, ultimately, the survival of juvenile seals. The SAM has often been hypothesized to affect foraging conditions for other aspects of this population, including the relative number of breeding females (van den Hoff et al., 2014), adult female foraging success (McMahon et al., 2017), and juvenile weaning mass (Clausius et al., 2017). In the Southern Ocean, SAM reflects different effects depending on latitude (Lovenduski & Gruber, 2005). Macquarie Island lies between the Polar (PF) and Subantarctic (SAF) fronts (Chapman et al., 2020). During a positive SAM, phytoplankton abundance increases south of the PF from additional iron upwelling (Lovenduski & Gruber, 2005). Westerly winds associated with positive SAM anomalies drive this productivity northward toward Macquarie Island via Ekman transport (Hall & Visbeck, 2002). In the process, elevated levels of primary productivity travel through the Southern Ocean food web resulting in increased abundance of elephant seal prey (fish and squid) in the area surrounding Macquarie Island the following year (Miller et al., 1985). The influence of SAM1 is greatest on juveniles, suggesting that localized prey availability is a major contributor to their survival. Juveniles stay relatively close to the breeding grounds when going on their first foraging trips (McConnell et al., 2002), while older juveniles and breeding adults are able to adapt to a potential lack of prey locally by adjusting their behavior and moving to alternate and more distant foraging sites (Guinet et al., 2014). Although older juveniles have a similar foraging range to adults, they are restricted by returning to haul out mid‐year back on Macquarie Island (Hindell & Burton, 1988a), requiring them to spend more time feeding in waters adjacent to the island (Hindell et al. under review). Furthermore, juveniles are generally less resilient to shortages in prey availability due to their smaller body size and lower blubber reserves (Oosthuizen et al., 2019). SAM1 may also influence first‐year survival by mediating maternal condition during gestation (McMahon et al., 2015). This may also explain the lagged effect on juvenile survival due to foraging success during the mother's pregnancy, reflecting resource acquisition before pups are weaned. Weaning success and first‐year survival are dependent on the mother's condition and foraging success during the critical pup rearing period (McMahon et al., 2000, 2015, 2017). However, this effect would only apply to first‐year seals and not the older juvenile states in our model.

In contrast, the SOI had a direct influence on the survival of these seals, suggesting it was affecting the abundance or availability of prey already present in the system. The continuous population decrease at Macquarie Island may be due to lower foraging success of breeding females whereby winter sea ice extent restricts access to productive shelf waters during the most critical time of resource acquisition (Clausius et al., 2017). Our survival estimates support this conclusion and provide another mechanism for the negative population growth rate at Macquarie Island. Other key foraging areas for this population are off the East Antarctic continental shelf in Victoria Land and the Ross Sea (Hindell et al., 2017b; McMahon et al., 2017). While warm cycle ENSO events (El Niño years) are associated with increased sea ice over the Ross Sea (Arrigo & van Dijken, 2004), the foraging area off the Victoria Land coast is subject to changes in wind stress, higher surface temperatures, and increased melting during El Niño events (Kwok & Comiso, 2002; Simpkins et al., 2012). Importantly, ENSO events are providing access to the East Antarctic continental slope for longer during the critical winter and spring foraging trips (de Little et al., 2007). Conversely, the extensive winter sea ice cover in this area during La Niña years would prevent access to this key foraging habitat (Hindell et al., 2017b; Simpkins et al., 2012). It is likely that in addition to SOI, the SAM also has a direct influence on the sea ice at the East Antarctic continental shelf (Simpkins et al., 2012), but our most parsimonious model only included the effects of a lagged SAM1.

In conclusion, the survival and demography of female southern elephant seals at Macquarie Island are influenced by climate indices of SAM and SOI directly, through changes in sea ice conditions preventing access to foraging areas, and indirectly by altering prey availability and productivity locally. How marine predators respond to these broad‐scale climate modes is important because it extends our understanding of biological responses to climate change, with short‐term anomalies giving an insight into future conditions (Trathan et al., 2007). Studying the demography of predator populations is a key component of assessing ecosystem change in the Southern Ocean (Weimerskirch et al., 2003).

Other Southern Ocean predators have shown demographic responses to SAM and SOI including wandering albatross (Fay et al., 2015), Adélie penguins (Wilson et al., 2001), and Weddell seals (Proffitt et al., 2007). Advanced ecological modeling enables an understanding of the complex interactions between external climatic influences and biological processes that drive animal population dynamics (Boyce et al., 2006). Species‐specific demographic changes give valuable insights into the overall ecosystem response to climate change, especially in regions where major shifts in food web dynamics are expected (Constable et al., 2014).

## CONFLICT OF INTEREST

None declared.

## AUTHOR CONTRIBUTIONS

**Sophia Volzke:** Conceptualization (equal); data curation (equal); formal analysis (equal); investigation (equal); methodology (equal); project administration (equal); visualization (equal); writing–original draft (equal); writing–review and editing (equal). **Clive R. McMahon:** Conceptualization (equal); data curation (equal); funding acquisition (equal); investigation (equal); methodology (equal); project administration (equal); resources (equal); supervision (supporting); writing–review and editing (equal). **Mark A. Hindell:** Conceptualization (equal); funding acquisition (equal); investigation (equal); methodology (equal); project administration (equal); resources (equal); supervision (supporting); validation (equal); writing–review and editing (lead). **Harry R. Burton:** Conceptualization (equal); funding acquisition (equal); writing–review and editing (supporting). **Simon J. Wotherspoon:** Conceptualization (equal); data curation (equal); formal analysis (equal); investigation (equal); methodology (equal); project administration (equal); resources (equal); software (equal); supervision (lead); validation (equal); visualization (equal); writing–review and editing (supporting).

### OPEN RESEARCH BADGES

This article has earned an Open Data Badge for making publicly available the digitally‐shareable data necessary to reproduce the reported results. The data is available at https://metadata.imas.utas.edu.au/geonetwork/srv/eng/metadata.show?uuid=94f859d3‐922b‐4fdd‐b6e9‐3a6186477bdc.

## Supporting information

Appendix S1

Appendix S2

## Data Availability

Macquarie Island southern elephant seal mark–recapture data are openly available via Dryad https://doi.org/10.5061/dryad.zpc866t7f.
