# Supplementary material for: Climate influences on female survival in a declining population of southern elephant seals (Mirounga leonina)
Source: Ecol Evol. 2021 Jul 27;11(16):11333–44. doi: 10.1002/ece3.7919 (PMC8366891; doi:10.1002/ece3.7919)

## Appendix S1: Model preparation

This includes data reporting (number of individuals and their time of tagging) and model testing (correlation and overdispersion tests).

## Appendix S1A: Tag Data

The total number of individuals: 9690

Illustration of the increasing number of individuals (nseal) over the relevant time period:


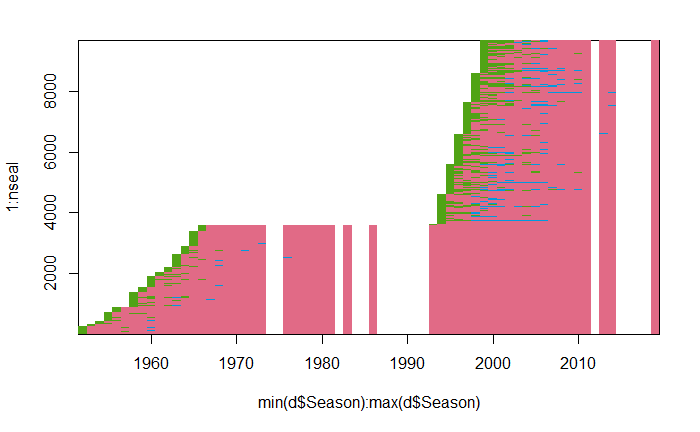


Histogram of the number of seals tagged as pups (first) during the relevant time periods.


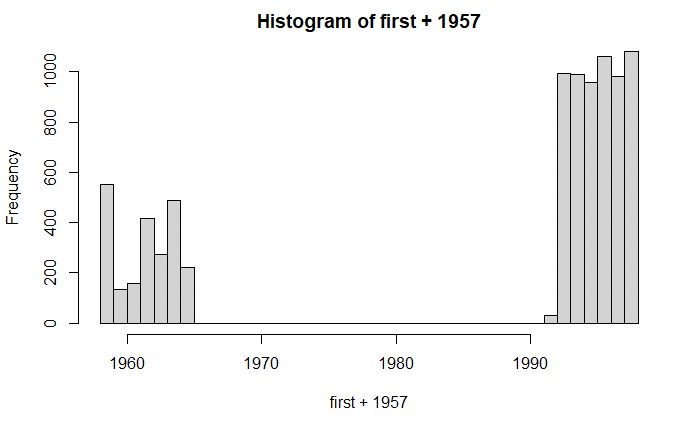


## Appendix S1B: Correlation testing

**Annual averages** of SAM & SOI from 1957-2019

ggscatter(d.Annual, x = "SAM", y = "SOI",

add = "reg.line", conf.int = TRUE,

cor.coef = TRUE, cor.method = "pearson",

xlab = "Annual SAM", ylab = "Annual SOI")


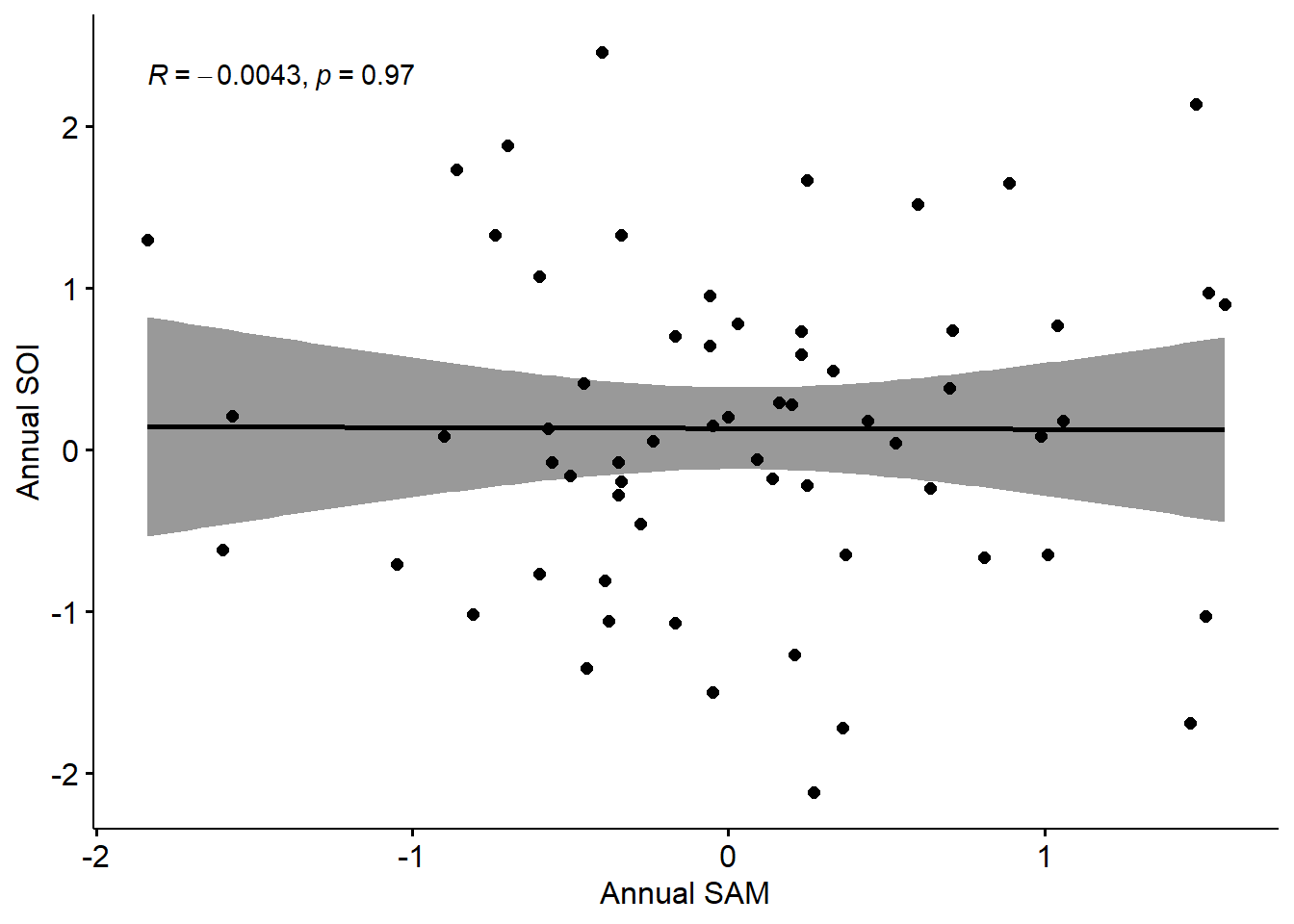


**Seasonal data** (compared only for autumn averages of each year from 1957-2019)

- AUT = September + October + November averages
- This is the most relevant time when most female seals are ashore

**library**("ggpubr")

ggscatter(d.Seasonal, x = "Seasonal_SAM", y = "Seasonal_SOI",

add = "reg.line", conf.int = TRUE,

cor.coef = TRUE, cor.method = "pearson",

xlab = "Seasonal SAM", ylab = "Seasonal SOI")


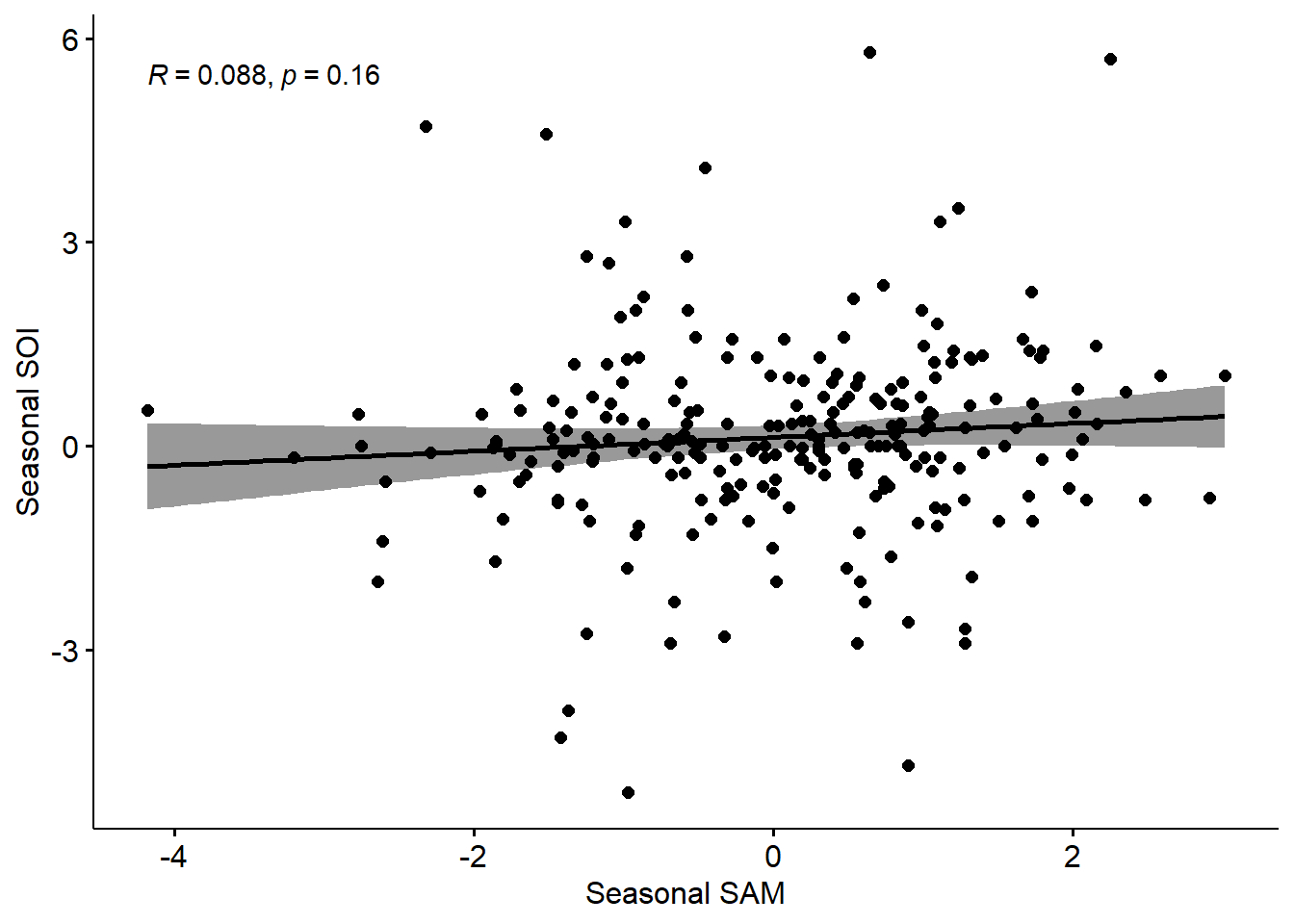


**Monthly data**

- Monthly Sam and SOI averages from 1957-2019

**library**("ggpubr")

ggscatter(d.Monthly, x = "Monthly_SAM", y = "Monthly_SOI",

add = "reg.line", conf.int = TRUE,

cor.coef = TRUE, cor.method = "pearson",

xlab = "Monthly SAM", ylab = "Monthly SOI")


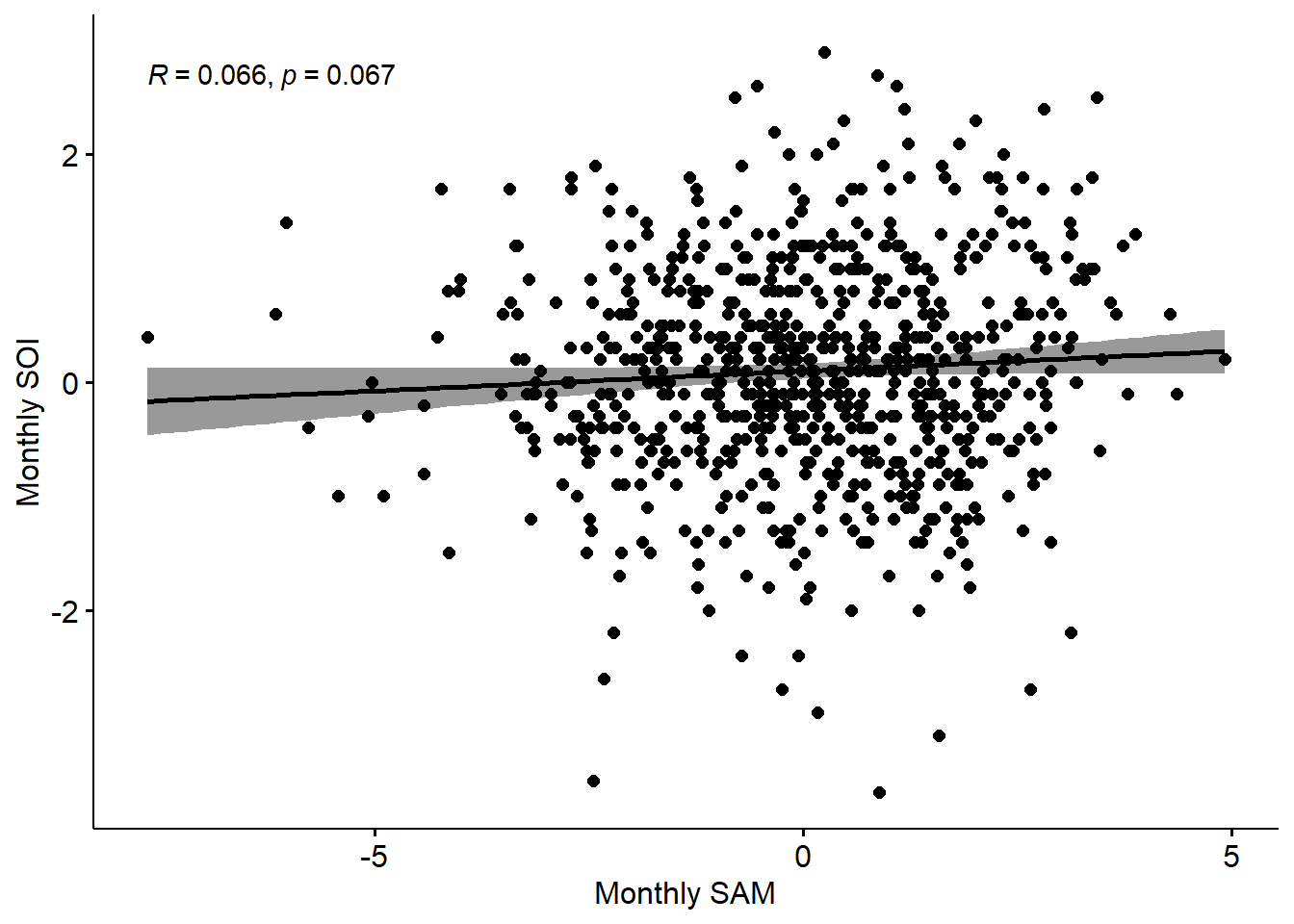


## Appendix S1C: Checking for Overdispersion

Comparing the distribution of simulated likelihoods from parametric bootstrap with the observed likelihoods.

load("parboot.RData")

ps <- unlist(lapply(ps,**function**(fit) fit$logL))

hist(ps)

abline(v=pars$logL)


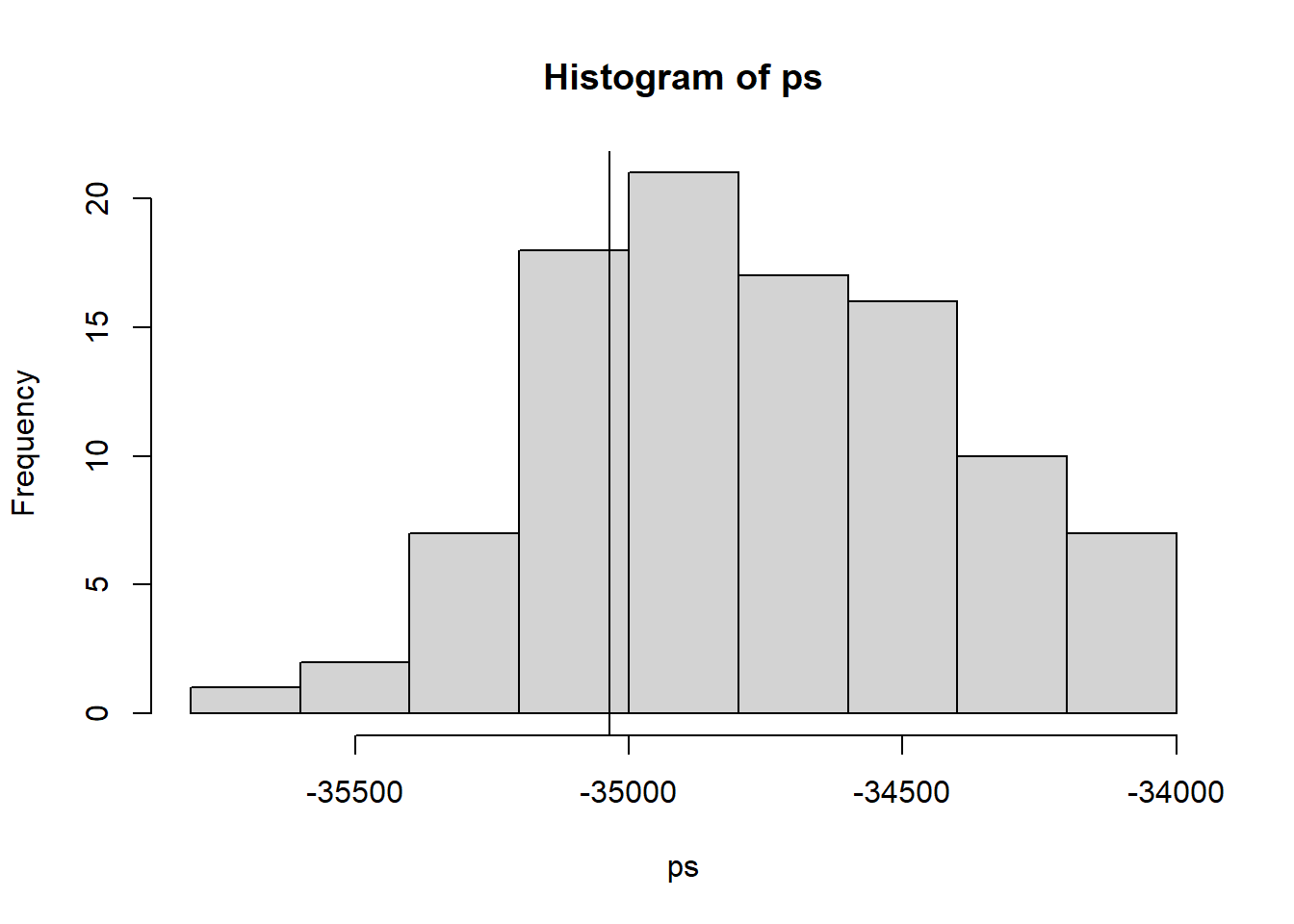

Supplement: Supplementary file 1 — Appendix S1 [file ECE3-11-11333-s001.docx]
