# Supplementary material for: Climate influences on female survival in a declining population of southern elephant seals (Mirounga leonina)
Source: Ecol Evol. 2021 Jul 27;11(16):11333–44. doi: 10.1002/ece3.7919 (PMC8366891; doi:10.1002/ece3.7919)
Supplement: Supplementary file 2 — Appendix S2 [file ECE3-11-11333-s002.docx]

## Appendix S2: Additional Results

For all these graphs, the time displayed on the x-axis starts at the first year of our capture history
t = 0 = 1957 and progresses from there, for example t = 10 = 1957 + 10 years

### Weights (*W*)

The expected numbers in each state by year:


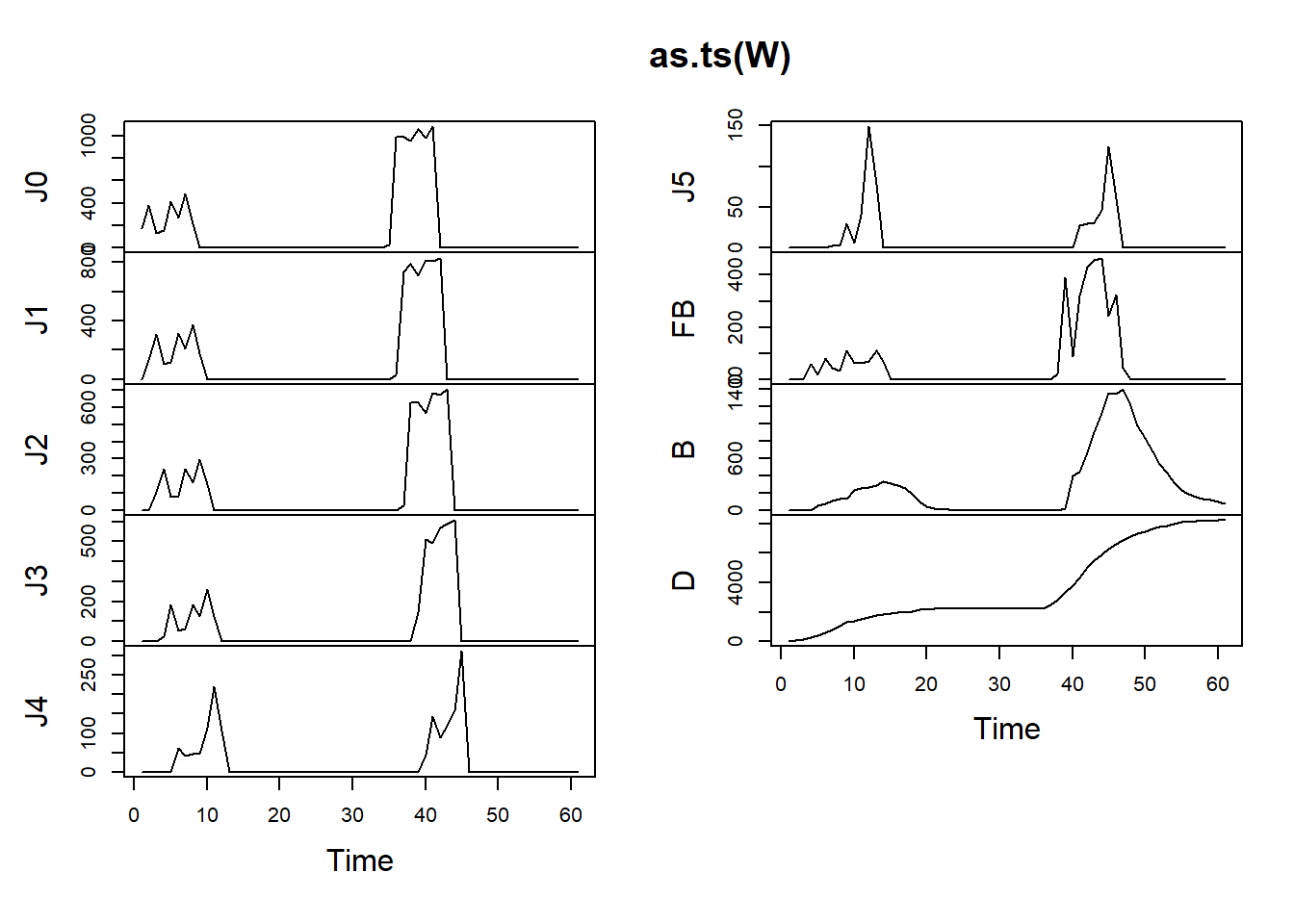


These ‘weights’ are used as the basis of our Figure 2 & 3 outline shade to indicate the amount of underlying data that is informing the modelled estimates at any given time throughout the study period.

### Emission (*dx*)

**SUMMARY** - The fitted emission probabilities (average):
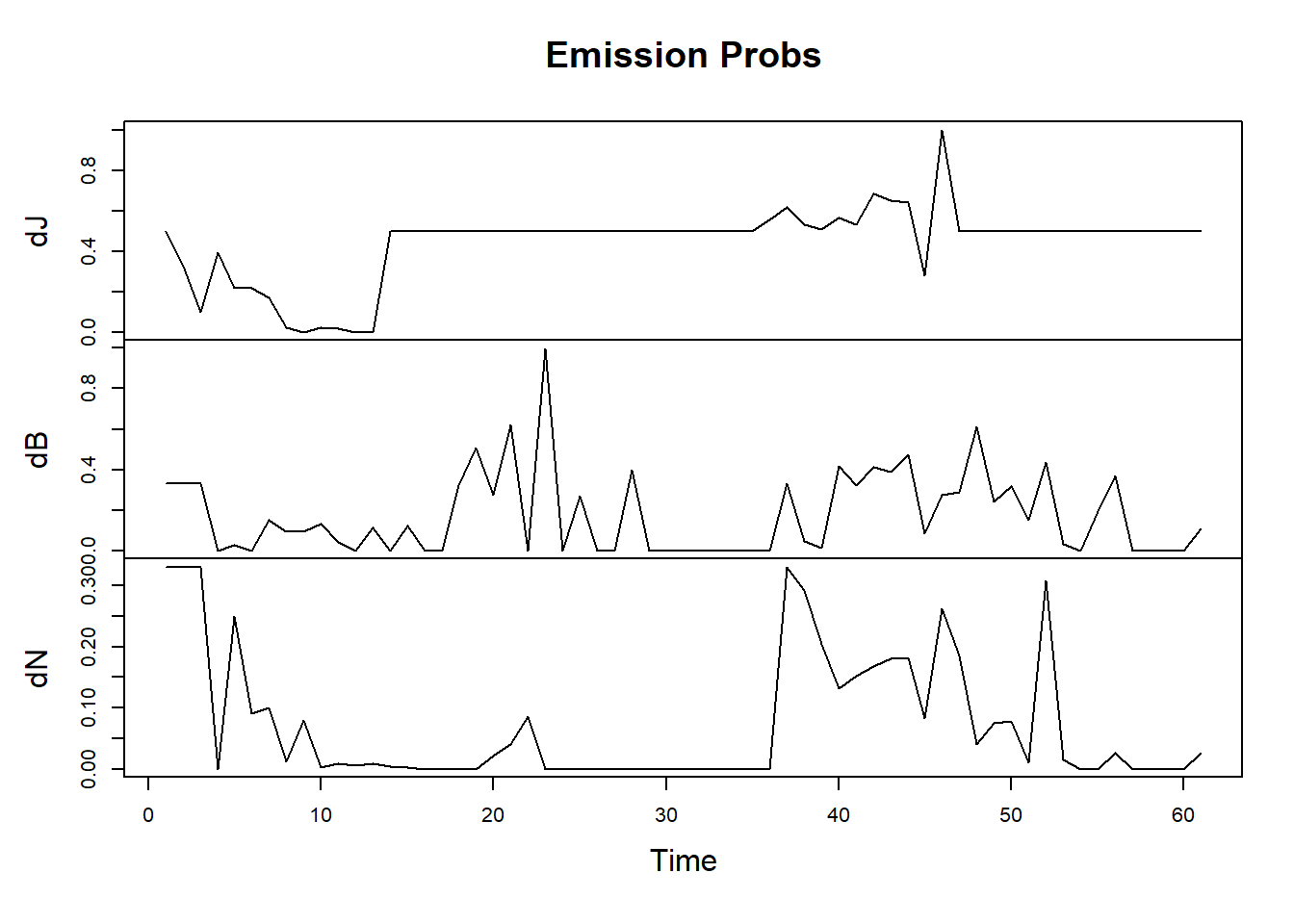


Emission probabilities describe the probability of being detected, as per emission matrix (Figure 1).

**
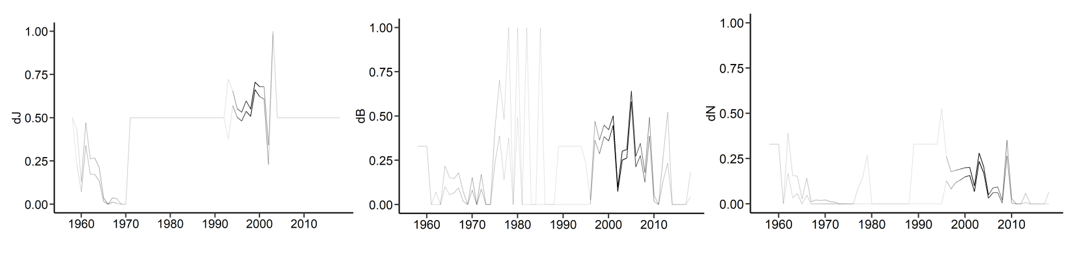
DETAILED** - Broken down into lower and upper quantiles with weighted outline:

The 1990s clearly had higher detection rates, especially for juveniles (dJ), but also for breeding (dB) and non-breeding adults (dN). It should be noted that the 1990s recapture study was set out to investigate the demography during early-life stages (pup and first-year survival) specifically (e.g. McMahon, Burton & Bester 1999). Therefore, these results reflect the 1990s as being the more intensive study period in terms of resight effort, which is entirely consistent with our modelled findings of having more precise results and larger weights (darker outlines) in the 1990s compared to the 1960s.

### Maturation (*Mx*)

**SUMMARY** - The fitted maturation probabilities (average)


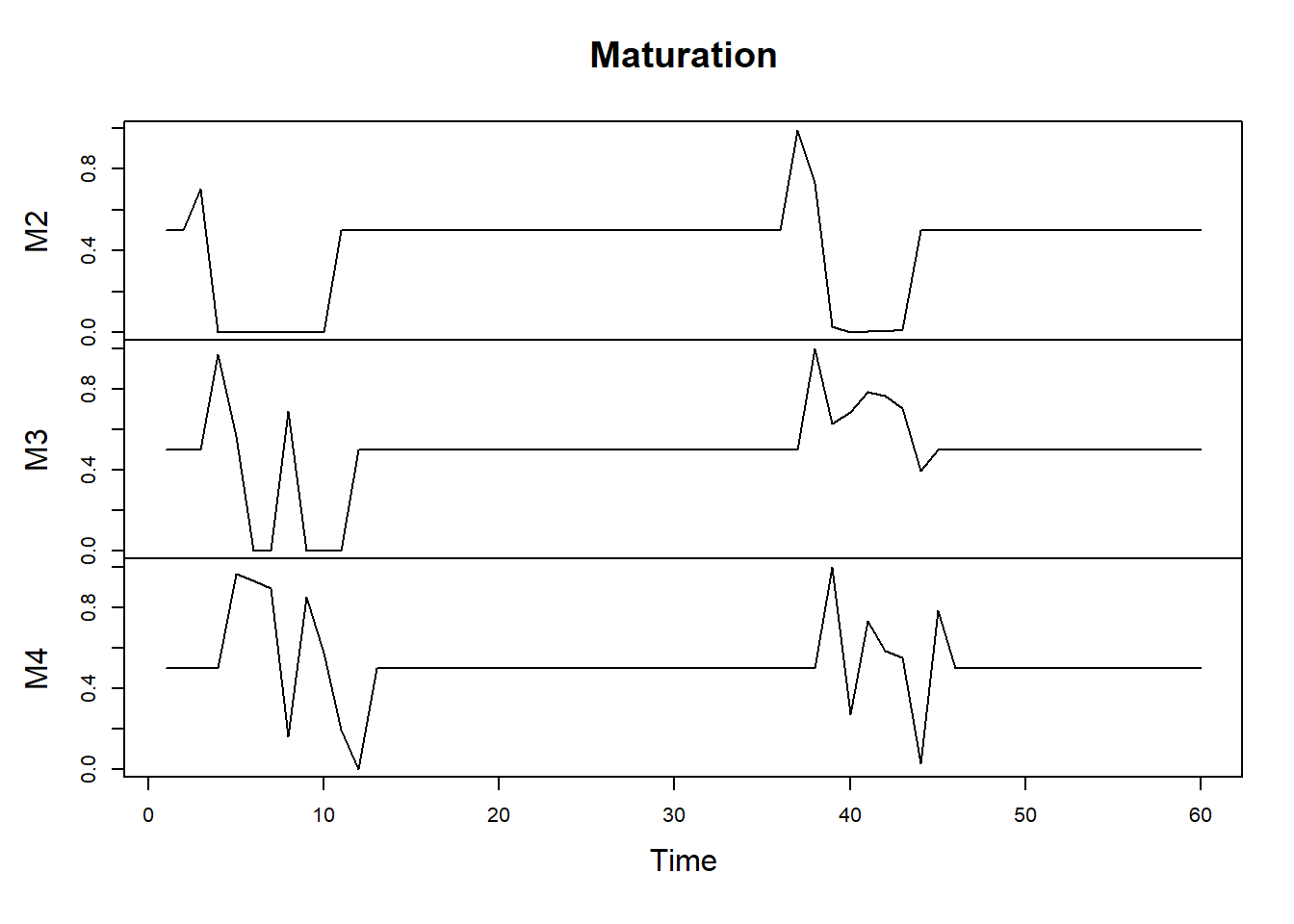


Maturation describes the probability for an individual to transition to the first-time breeder state the following year. This is defined by being detected with a pup during the breeding season (September to October).
